# Prognosis of Breast Cancer in Women in Their 20s: Clinical and Radiological Insights

**DOI:** 10.3390/diagnostics15162072

**Published:** 2025-08-19

**Authors:** Inyoung Youn, Eun Young Ko, Jeong Eon Lee, Boo-Kyung Han, Eun Sook Ko, Ji Soo Choi, Haejung Kim, Myoung Kyoung Kim, Mi Yeon Lee, Suhyeon Moon, Mi-ri Kwon

**Affiliations:** 1 Department of Radiology, Kangbuk Samsung Hospital, School of Medicine, Sungkyunkwan University, 29 Saemunan-ro, Jongno-gu, Seoul 03181, Republic of Korea; iy.youn@samsung.com (I.Y.); miri.kwon@samsung.com (M.-r.K.); 2 Department of Radiology and Center for Imaging Science, Samsung Medical Center, School of Medicine, Sungkyunkwan University, Seoul 06351, Republic of Korea; bookyung.han@samsung.com (B.-K.H.); es.ko@samsung.com (E.S.K.); jisoo.choi@samsung.com (J.S.C.); haejung220.kim@samsung.com (H.K.); myoungkyoung.kim@samsung.com (M.K.K.); 3 Division of Breast Surgery, Department of Surgery, Samsung Medical Center, School of Medicine, Sungkyunkwan University, Seoul 06351, Republic of Korea; 4 Division of Biostatistics, Department of Academic Research, Kangbuk Samsung Hospital, 29 Saemunan-ro, Jongno-gu, Seoul 03181, Republic of Korea; my7713.lee@samsung.com (M.Y.L.); suhyeon71.moon@samsung.com (S.M.)

**Keywords:** prognosis, breast neoplasms, young women, epidemiology, radiology

## Abstract

**Background/Objectives:** We analyzed clinical and radiological characteristics and prognostic factors specific to young patients with breast cancer (YBC) aged <30 years. **Methods:** This retrospective study included 132 women aged <30 years who underwent breast surgery between 2008 and 2013. The clinical and radiological findings of the patients were examined and compared according to recurrence or death status at follow-up. Disease-free survival (DFS) and overall survival (OS) rates were also assessed. **Results:** Most patients (mean age, 27.1 years) presented with palpable lesions (85.6%). Hormone receptor-positive/human epidermal growth factor receptor-negative cancer was the most common molecular subtype (59.8%), followed by triple-negative breast cancer (28.0%), with high Ki-67 expression (62.1%). Mammography and ultrasound detected abnormalities in 90.1% and 97.3% of patients, respectively, whereas magnetic resonance imaging detected abnormalities in all patients. During the follow-up period (8–10 years), 28.5% of the patients experienced recurrence and 11.5% died. The calculated DFS and OS at 5 years were 80.8% and 69.8% and 91.3% and 87.8% at 10 years, respectively. Statistically significant factors associated with DFS/OS included the *BRCA1* gene mutation, with preoperative neoadjuvant chemotherapy, no hormone therapy, larger tumor size, negative hormone receptor status, high Ki-67 expression, and some radiological findings, including asymmetry with calcifications on mammography, no sonographic echogenic rind of mass, and mild vascularity on Doppler study. **Conclusions:** Our study highlights the aggressive nature of breast cancer in YBC aged <30 years, with relatively high rates of recurrence and mortality. Significant factors affecting prognosis may guide personalized treatment approaches and predict the prognosis.

## 1. Introduction

Breast cancer (BC) is rare in young women aged <30 years, with a reported incidence of 0.65% of all BC cases in Western countries compared to >3.1% reported in Asian countries [1]. Studies have indicated that young patients with BC (YBC) often exhibit aggressive pathological features, highlighting the necessity for a more nuanced approach to treatment planning and prognosis prediction [2,3]. However, early diagnosis is challenging because the screening program does not include the population of YBC, and the dense breast tissue in this age group is likely to decrease lesion palpability during the early period of the disease [4]. Moreover, young age itself is a major independent risk factor for BC recurrence [3,5].

These distinct characteristics of YBC require a thorough investigation of their clinical, radiological, and prognostic factors to tailor effective management strategies [6,7]. Furthermore, the interplay between clinical and radiological factors in this cohort remains a pivotal yet underexplored area, warranting a comprehensive investigation to delineate its unique characteristics. Recently, the European School of Oncology and the European Society of Medical Oncology published guidelines on YBC to establish an international consensus [2].

To the best of our knowledge, few studies have focused on the clinical and radiological features of YBC and the correlation between these features and prognosis in patients aged <30 years [1,2,6,7,8,9,10]. Most previous studies on YBC have included patients with BC in their 30s, and their sample sizes were relatively small when using follow-up data over a relatively short period. Therefore, this study aimed to analyze the clinical and radiological characteristics and prognostic factors specific to BC in individuals aged <30 years.

## 2. Materials and Methods

The institutional review board approved this single center retrospective study (IRB No. 2023-12-032-001). The requirement for informed consent was waived for the retrospective reviewing clinical images and medical records.

### 2.1. Patients

From January 2008 to December 2013, of the 8843 women who underwent BC surgery at our institution, 145 (1.6%) were aged <30 years. We excluded the following patients: (1) those without detailed clinical/pathological information (n = 4) and (2) those with tumors of mesenchymal origin, such as malignant phyllodes tumors or angiosarcomas, following BC surgery (n = 9). A total of 132 patients aged <30 years were included in the clinical analysis. Among them, patients with BC confirmed by excisional (n = 13) or vacuum-assisted (n = 7) biopsies without available images representing BC were included in the analysis of clinical factors but were excluded from the radiological analysis. Finally, 112 patients were included in the radiological analysis.

### 2.2. Analysis of Basic Clinical with Histopathological Characteristics

The patients’ medical records were reviewed, and clinical data were collected as follows: (1) at the time of diagnosis and surgery: age; body mass index (BMI); family history; presence of a *BRCA* gene mutation; metastasis to other organs; response to neoadjuvant chemotherapy (NAC); and date and methods of surgery to the breast and axilla; and (2) follow-up data after surgery: type of therapy after surgery, including radiation therapy (RTx), chemotherapy (CTX), and hormone therapy (HRT), and prognostic variables, such as time to events of first recurrence or survival, site of recurrence (local, metachronous contralateral breast [more than 6 months following the detection of first BC], or distant metastasis [bone, lung, brain, liver, or other organs]), and reason for death.

Histopathological results were analyzed based on the pathology report after BC surgery, including histopathology, size, multiplicity, lymphovascular invasion (LVI), extensive intraductal component (EIC), nipple–areolar complex involvement, histological grade, and surgical staging. In patients who underwent NAC, the clinical stage was evaluated before NAC instead of the surgical stage.

The estrogen receptor (ER), progesterone receptor (PR), human epidermal growth factor receptor 2 (HER2), and Ki-67 index were evaluated. Hormone receptor (HR) positivity was defined as positive ER or PR expression. The cutoff level of Ki-67 was established at 20% [11]. Based on the ER, PR, and HER2 status, the tumor subtypes were classified as follows: HR+/HER2−, HR+/HER2+, HR−/HER2+, and triple-negative BC (TNBC).

### 2.3. Radiological Analysis

Of the 112 patients with available preoperative imaging data, the imaging characteristics of mammography, ultrasound (US), and magnetic resonance imaging (MRI) were assessed through a retrospective review by two radiologists in consensus (*Blindedfor Submission*) according to the ACR BI-RADS lexicon [12,13,14,15]. The assessments were made jointly rather than independently.

For mammography, we assessed breast composition and analyzed the features of each lesion as follows: (1) mass: shape (oval/round, irregular), margin (circumscribed, not circumscribed), and density (hyper, iso, low); (2) calcification: distribution (segmental, grouped, regional, diffuse) and shape (fine linear/pleomorphic, coarse heterogeneous, amorphous); and (3) asymmetry. Combined architectural distortion was also reported.

For US, we assessed background echotexture [12] and analyzed US features of the lesions as follows: (1) mass: shape (oval, round, irregular), orientation (parallel, nonparallel), margin (circumscribed, not circumscribed), echogenicity (hypo, iso, hyper, anechoic, complexed cystic and solid, heterogenous), and echogenic rind; (2) non-mass lesions: distribution (focal, linear/segmental, regional), echogenicity (hypo, iso, hyper, anechoic), and intralesional cysts; and (3) associated findings, including calcifications within the lesions, architectural distortion, ductal changes, and posterior features (no, enhancement, shadowing). If available, we reviewed their Doppler features and divided them into avascular, mild (1–2 dots), and hypervascular (≥3 dots, branching or penetrating vessels within the lesion).

For MRI, background parenchymal enhancement and lesion type were evaluated. The MRI features of each lesion were evaluated as follows: (1) mass: shape (oval/round, irregular), margin (circumscribed, not circumscribed), enhancement pattern (homogenous or heterogeneous), rim enhancement, and T2 signal intensity; and (2) non-mass enhancement: distribution (focal, segmental, regional, diffuse) and enhancement pattern (homogenous, heterogeneous). Enhancement kinetics on dynamic contrast-enhanced MRI (persistent, plateau, washout) were evaluated.

### 2.4. Statistical Analysis

Continuous variables are reported as the mean ± standard deviation or median value and were analyzed using the independent *t*-test or Wilcoxon-rank sum test. Categorical variables are reported as percentages with frequency and were evaluated using Pearson’s chi-square or Fisher’s exact tests. Missing data were not imputed, and all statistical analyses were performed using only the available case data. We divided the patients into two groups according to their recurrence or death status at follow-up and compared their clinical and radiological factors. Patients with metastasis at the time of diagnosis were excluded from the evaluation of follow-up outcomes and the prognostic factors.

Disease-free survival (DFS) and overall survival (OS) were also calculated. DFS was defined as the time from the date of diagnosis to the date of the first episode of recurrence. OS was defined as the time from diagnosis to death. Disease relapse and survival were estimated using Kaplan–Meier plots to visualize the survival probability. To estimate the prognostic clinical and radiological factors, we used Cox proportional hazards models and calculated hazard ratios with 95% confidence intervals (CIs) using forest plots. All statistical analyses were performed using IBM SPSS software v.29 for Windows (IBM Corp., Armonk, NY, USA). Statistical significance was defined as *p* < 0.050.

## 3. Results

### 3.1. Clinical and Pathological Characteristics of the Patients and Tumors

The basic characteristics of the patients assessed in this study are summarized in Table 1. The mean age of the 132 YBC at the time of diagnosis was 27.1 ± 1.9 years (range, 19–29 years). All patients were premenopausal, and 113 (85.6%) had palpable lesions. Only 19 patients (14.4%) had a family history of BC. *BRCA* gene mutation tests were performed in 54 patients (40.9%), of whom 11 (20.4%) showed positive results. Two patients (1.5%) had metastases to other organs at the time of diagnosis, and they underwent surgical treatment after completing palliative CTX. NAC was performed in 27 patients (20.5%), and a pathologic complete response (pCR) rate of 14.8% (4/27) was achieved. Most patients (75.0%) underwent breast-conserving surgery. None of the patients in this study had a positive margin at the time of surgery.

The histopathological findings are presented in Table 2. The mean tumor size was 3.5 ± 2.4 cm (range, 0.6–12 cm). The most common histopathological result was invasive ductal carcinoma (79.5%), followed by ductal carcinoma in situ (9.1%). Two patients had more than one tumor type. Multiplicity (22.0%), LVI (43.8%), EIC (35.1%), and nipple–areolar complex involvement (6.8%) were frequently observed, and the rate in the high Ki-67 group (>20%) was 62.1% (82/132). TNBC accounted for 28.0%, whereas the HR+/HER2− subtype was observed in 59.8% of cases.

### 3.2. Radiological Characteristics

All patients underwent mammography, breast US, and MRI at diagnosis, except for three pregnant patients. Of these three patients, two underwent mammography, and all underwent breast US; however, none underwent breast MRI. Therefore, we evaluated 111 mammography, 112 US, and 109 MRI images and analyzed the radiological findings of BC in these patients (Table 3, Table 4 and Table 5).

**Figure 1:**
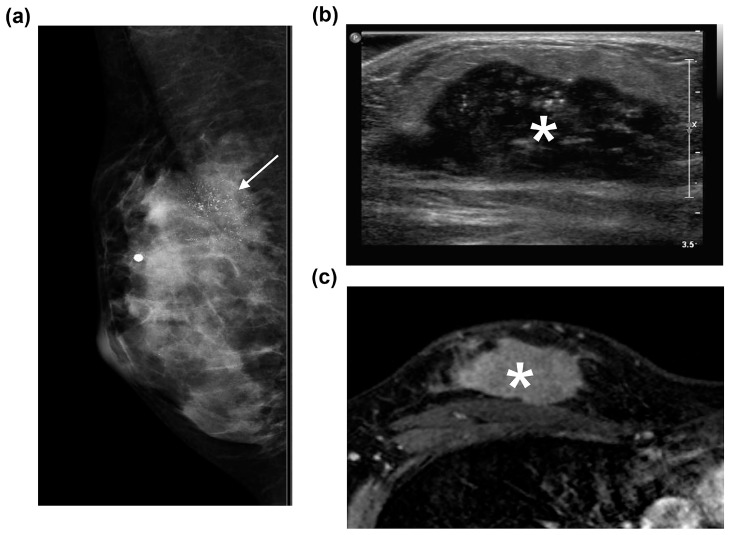
Imaging findings of ER+/PR+/HER2− invasive breast cancer of no special type with high Ki-67 expression in the right breast of a 17-year-old female. (**a**) Right mediolateral oblique view of the mammography showing a 5 cm, irregular, not circumscribed isodense mass with a segmental distribution, as well as fine, linear-branching suspicious calcifications (arrow) in the upper breast. (**b**) B-mode ultrasound image showing a large, irregular, not circumscribed, hypoechoic mass (asterisk) with an echogenic rind. (**c**) Axial contrast-enhanced fat-suppressed T1-weighted magnetic resonance imaging showing an irregular, non-circumscribed, heterogeneous, rim-enhancing mass (asterisk) with washout kinetics in the right upper outer quadrant of the breast. The patient underwent breast-conserving surgery with axillary lymph node dissection after two years of palliative chemotherapy for bone metastasis of the thoracolumbar spine at the time of diagnosis, followed by additional radiotherapy with hormone therapy. Three years after surgery, pleural metastasis with hilar lymph node metastasis was observed, and the patient died two years after chemotherapy. ER: estrogen receptor; PR: progesterone receptor; HER2: human epidermal growth factor receptor 2.

Although most of the patients presented with a dense breast composition (97.3%), 90.1% showed positive findings on mammography. Masses were observed in 78 patients (70.2%; irregular shaped [74.4%] and not circumscribed [89.7%]); calcifications were observed in 58 patients (52.2%); and 10 patients showed other findings (focal or global asymmetry). Among the 112 US examinations, the background echotexture was mostly homogenous-fibroglandular (83.9%). On US, 109 lesions (97.3%) showed positive findings, with a mass being the most common finding (80.4%). Although these were malignant tumors, round or oval-shaped masses were relatively frequent (44.3%). MRI revealed positive findings in all the cases. Masses were the most frequent positive finding (83.5%), mostly with a round or oval shape (55.0%), not circumscribed margin (90.1%), heterogeneous internal enhancement (71.4%), and rim enhancement (64.8%). Washout or plateau enhancement kinetics were observed in 93.6% of the cases (102/109; Figure 1).

### 3.3. Follow-Up Outcomes and Prognostic Factors

**Figure 2:**
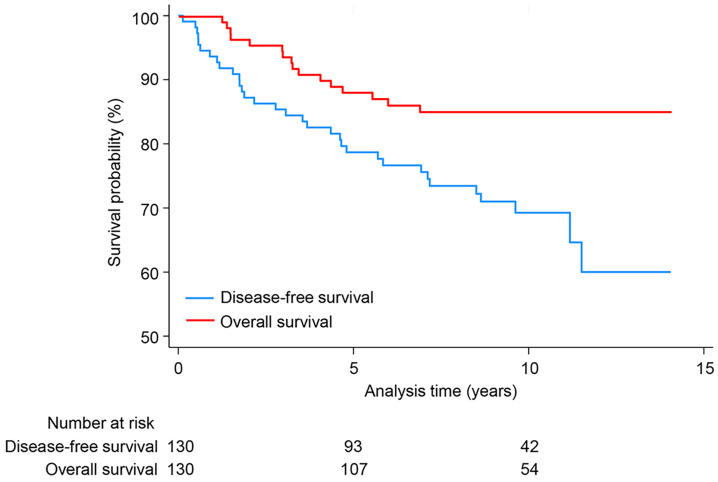
Disease-free survival (DFS; blue line) and overall survival (OS; red line) in young patients with breast cancer aged <30 years. 5-year DFS: 80.8% (95% confidence interval [CI], 74.2–88.1%); 10-year DFS: 69.8% (95% CI, 61.8–79.0%); 5-year OS: 91.3% (95% CI, 86.5–96.3%); 10-year OS: 87.8% (95% CI, 82.1–93.8%).

Two patients who received palliative CTX for metastasis at the time of diagnosis were excluded from the evaluation of follow-up outcomes and prognostic factor analysis. Consequently, 130 and 110 patients were evaluated for clinicopathological and radiological factors, respectively, in the prognostic analysis. During the follow-up period of the 130 enrolled patients, 37 (28.5%) experienced recurrence (21 local [16.2%; 10 ipsilateral breast, 1 mastectomy bed, and 10 axillary/supraclavicular LN], 7 metachronous contralateral breast [5.4%], and 19 distant metastases [14.6%]). Death due to BC occurred in 15 (11.5%) patients. The median follow-up period was 8.73 years (range, 0.04–14.05 years) for recurrence and 9.43 years (range, 0.04–14.12 years) for survival. The entire cohort showed a DFS and OS of 80.8% (95% CI, 74.2–88.1%) and 69.8% (95% CI, 61.8–79.0%) at 5 years and 91.3% (95% CI, 86.5–96.3%) and 87.8% (95% CI, 82.1–93.8%) at 10 years (Figure 2).

Table 6 summarizes the statistically significant factors associated with recurrence and death during the follow-up period. No significant differences were observed in the recurrence status across all clinical variables (*p* > 0.050), except for death at follow-up (*p* < 0.001; Table S1). For survival, several clinical variables displayed significant differences in expired patients, including a higher median BMI (*p* = 0.040), positive *BRCA1* gene mutation (*p* = 0.046), preoperative NAC treatment (*p* = 0.016), axillary lymph node dissection in the total mastectomy group (*p* = 0.044), less HRT (*p* = 0.018), with recurrence (*p* < 0.001), larger mean tumor size (*p* = 0.016), histologic grade of invasive cancer (*p* = 0.018), non-positive PR rate (*p* = 0.001), and high Ki-67 (*p* = 0.001). In the subgroup analysis according to imaging findings, only masses with no echogenic rind on US in the recurrence group (96.3% [26/27] vs. 40.0% [44/61], *p* = 0.009) were significant, and no statistical significance was observed in the other groups for either recurrence status or death (*p* > 0.050; Table S2).

**Figure 3:**
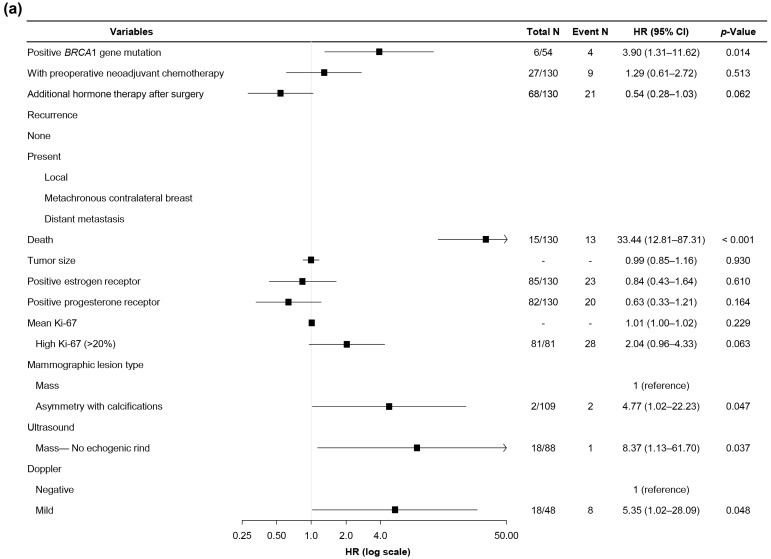

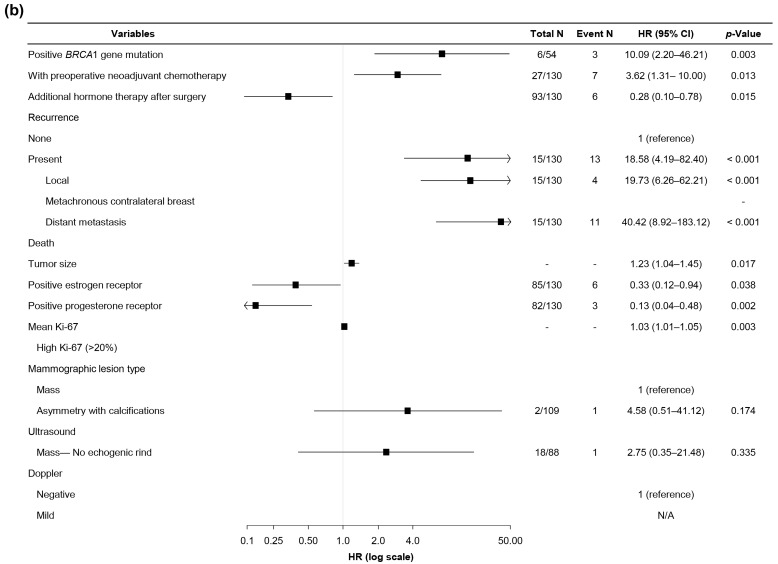
Forest plot of the hazard ratios (HRs) for factors associated with disease-free (**a**) and overall (**b**) survival. The plot shows the HRs with 95% confidence intervals (CIs) for each variable included in the analysis. Squares represent the point estimates of HRs, and horizontal lines indicate the 95% CIs. Variables with an HR > 1 suggest an increased risk, whereas an HR < 1 indicates a reduced risk compared with the reference group.

Table 7 and Figure 3 show the Cox proportional hazards regression analysis results with forest plots for DFS and OS. The factors with significantly higher hazard ratios for DFS were *BRCA1* gene mutation (3.90, *p* = 0.014), death at follow-up (33.44, *p* < 0.001), asymmetry with calcifications on mammography (4.77, *p* = 0.047), no echogenic rind of mass on US (8.37, *p* = 0.037), and mild vascularity on Doppler US (5.35, *p* = 0.048). The factors that significantly decreased OS were *BRCA1* gene mutation (10.09, *p* = 0.003), patients who underwent NAC (3.62, *p* = 0.013), no adjuvant HRT (0.28, *p* = 0.015), recurrence at follow-up (18.58, *p* < 0.001; local, 19.73, *p* < 0.001; distant, 40.42, *p* < 0.001), larger tumor size (1.23, *p* = 0.017), non-positive ER (0.33, *p* = 0.038), and non-positive PR (0.13, *p* = 0.002). The other variables showed no significant differences (*p* > 0.050; Tables S3 and S4).

## 4. Discussion

BC is the most common malignancy in women globally, with an estimated 2.3 million new cases reported in 2022 [16]. Notably, the incidence of YBC is relatively higher in Asia than in Western countries [1,17]. The absence of a standardized age definition for YBC poses a challenge, with studies employing various thresholds, such as <30 [1,9,18], <35 [5,7], and <40 years [2,6,8,10,17,19,20], as well as considering premenopausal status [21]. Alhaidary et al. [8] reported that the proportion of patients aged <30 years is approximately 16% among YBC aged <40 years. Our study addresses this knowledge gap by focusing on an under-represented cohort of YBC aged <30 years. Recognizing the distinct clinical, radiological, and prognostic features of this subgroup is crucial because patients with palpable lesions at this age are often mistakenly considered benign. In this study, the rate of BC in patients aged <30 years was 1.6% (145/8843), and most patients (85.6%, 113/132) presented with palpable lesions.

Numerous studies have highlighted the aggressive course of BC in YBC, characterized by a higher frequency of family history of BC, *BRCA* gene mutations, higher tumor grade, larger tumor size, lymph node involvement, distant metastasis at diagnosis, HR-negativity, higher Ki-67 levels, more LVI, and a higher TNBC rate, contributing to poorer outcomes [7,17,20,22,23,24,25,26]. Our study corroborates these findings, revealing a frequent positive family history (14.4%), *BRCA* gene mutations (20.4%), relatively large tumor size (3.5 cm), frequent multiplicity (22.0%), positive LVI (43.8%), negative-HER2 expression (87.9%), and a higher proportion of TNBC (28.0%) than those measured in previous reports of YBC under 35 or 40 years of age [17,23]. Notably, high Ki-67 expression of approximately 62.1% suggests increased proliferative activity, contributing to the aggressive nature of BC in YBC, as previously reported [27].

We observed that the imaging features of YBC were similar to those observed in older patients [1,8,10,18,23,28] despite our baseline age of 30 years. In our study, mammography exhibited a positive predictive value of 90.1%, even in the presence of dense breasts, and an irregular mass was the most common finding, consistent with previous studies [1,8,10,18,23,28]. US has emerged as the preferred initial imaging modality for YBC, particularly in patients aged <30 years, owing to its lack of radiation exposure and efficacy in imaging dense breasts. US detected 97.3% of YBC cases in our study, with all mammography-negative lesions exhibiting positive US results (nine masses and two non-mass lesions), consistent with previous studies [1,18,28]. MRI is the most sensitive modality for evaluating BC and may play a significant role in selecting treatment options and evaluating the extent of the disease [29]. In our study, MRI detected all lesions as predominantly not circumscribed, heterogeneous, rim-enhancing masses, which is consistent with previous studies [1,10,23,28].

Previous studies have reported lower 5- and 10-year OS rates of 86.4% and 69.0–71.1%, respectively, for YBC <35–40 years of age, which are worse than those of older age groups, particularly in lower tumor stages and Luminal B tumors [7,20,26,30]. Our study revealed 5- and 10-year OS rates of 91.3% and 87.8%, respectively, which are in good agreement with those of previous YBC cohorts. The relatively higher OS observed in our study may be attributed to several factors, including ductal carcinoma in situ (9.1%), increased rates of adjuvant RTx, lower histologic grade, and lower Ki-67 expression compared with those of other studies [7,20]. Moreover, we excluded cases with metastatic disease at diagnosis to evaluate the follow-up outcomes and prognostic factors. Further randomized controlled trials are warranted to accurately compare YBC across different age groups.

The recurrence and mortality rates in our cohort of patients aged <30 years without metastasis at the time of diagnosis were 28.5% and 11.5%, respectively, over a median follow-up period of 8.7 and 9.4 years. These rates were higher than those reported by Larson et al., who evaluated YBC aged <40 years and found lower recurrence and mortality rates (17.4% and 8.0%, respectively) during a similar follow-up period [31]. Our study identified several significant clinicopathological factors associated with recurrence and death, including higher BMI, *BRCA1*-positivity, preoperative NAC, positive ALND in total mastectomy, non-HRT after surgery, larger tumor size, poor histological grade, PR-negativity, and high Ki-67 expression. Moreover, the calculated HR of the above characteristics were significant for OS, except for BMI, method of surgery, and histologic grade. These factors serve as important surrogate markers of aggressive BC and underscore their relevance to YBC, which is consistent with previous studies [3,6,7,24,25].

Although radiological evaluation provides valuable insights into tumor characteristics, its correlation with prognosis, including DFS and OS, in the overall population without considering age, remains controversial [32]. Additionally, only a few studies report on the prognostic factors based on radiological findings in YBC [6,10,29,33]. In our study, the mammographic asymmetry with calcifications (1.8%, 2/109) and absence of a US-detected echogenic rind in the mass (79.5%, 70/88) were significant factors associated with recurrence, although they were significantly associated with DFS only and not with OS. Further research is warranted to elucidate the prognostic value of the radiological findings in YBC.

Our study had some limitations. First, our study was retrospective, which may limit the generalizability of the results to prospectively evaluate the prognosis of YBC aged <30 years. Second, the relatively small sample size may have affected the statistical power. Third, the median follow-up period was 8.7 years; however, the absence of long-term follow-up data > 10 years may have underestimated the recurrence and mortality rates. Fourth, the absence of a standardized age definition for YBC poses a challenge when comparing our results with those of previous studies. Furthermore, comparisons between patients aged <30 and >30 years would provide valuable insights; however, this was not within the scope of this study because of the complexity of the data. Future research should address these comparisons to obtain more meaningful results. Despite these limitations, our study highlights the need for further research to understand the unique characteristics of YBC aged <30 years and for more careful examination and treatment of patients with factors that affect prognosis.

In conclusion, our study highlights the aggressive nature of BC in YBC aged <30 years, which is characterized by high TNBC rates and Ki-67 expression levels, as well as relatively high recurrence and mortality rates. The identification of these high-risk factors may aid in the development of personalized treatment strategies. In particular, patients with aggressive tumors may benefit from intensive adjuvant treatment and close clinical surveillance. These findings underscore the need for tailored management approaches in this challenging population.

## Figures and Tables

**Table 1:** Basic characteristics of the 132 young patients with breast cancer assessed in this study.

Characteristics	Value
Age (years)	27.1 ± 1.9
Body mass index (kg/m^2^)	21.4 ± 3.1
With family history	19/132 (14.4%)
*BRCA* mutation test ^a^	
Positive	11/54 (20.4%)
*BRCA1*	6/54 (11.1%)
*BRCA2*	5/54 (9.3%)
Negative	43/54 (79.6%)
Distant metastasis at the time of diagnosis ^b^	2/132 (1.5%)
pCR after neoadjuvant chemotherapy ^a^	4/27 (14.8%)
Method of surgery	
Breast-conserving surgery ^b^	99/132 (75.0%)
Without ALND	60/99 (60.6%)
With ALND ^b^	39/99 (39.4%)
Total mastectomy	33/132 (25.0%)
Without ALND	16/33 (48.5%)
With ALND	17/33 (51.5%)
Additional therapy after surgery	
Radiation therapy ^c^	101/132 (76.5%)
Chemotherapy	75/132 (56.8%)
Hormone therapy ^b^	91/132 (68.9%)
Recurrence after surgery	
Local ^c^	22/132 (16.7%)
Metachronous contralateral breast	7/132 (5.3%)
Distant metastasis ^b^	21/132 (15.9%)
None	93/132 (70.5%)
Survival	
Death ^b^	17/132 (12.9%)
Survived	115/132 (87.1%)

Data are presented as mean ± standard deviation or number/total number (percentage); pCR: pathologic complete response; ALND: axillary lymph node dissection. ^a^ Calculated among the available examinations. ^b^ Included two patients with metastasis observed at the time of diagnosis. ^c^ Included one of the two patients with metastasis observed at the time of diagnosis.

**Table 2:** Histopathological results of 132 young patients with breast cancer.

Variables	Value
Histopathological result ^a^	
Invasive ductal carcinoma, no special type ^b,d^	105/132 (79.5%)
Ductal carcinoma in situ	12/132 (9.1%)
Mucinous carcinoma ^c^	7/132 (5.3%)
Metaplastic carcinoma ^b^	4/132 (3%)
Papillary carcinoma ^c^	2/132 (1.5%)
Invasive lobular carcinoma	2/132 (1.5%)
Medullary carcinoma	1/132 (0.8%)
Secretary carcinoma	1/132 (0.8%)
With multiplicity ^e^	29/132 (22%)
With lymphovascular invasion ^e,f^	53/121 (43.8%)
With extensive intraductal component ^f^	39/111 (35.1%)
With nipple–areolar complex involvement	9/132 (6.8%)
Histologic grade of invasive cancer ^f^	
Well	24/109 (22.0%)
Moderate ^e^	46/109 (42.2%)
Poor ^e^	39/109 (35.8%)
Surgical staging	
Stage 0	12/103 (11.7%)
Stage 1	40/103 (38.8%)
Stage 2	44/103 (42.7%)
Stage 3–4	7/103 (6.8%)
29 lesions with chemotherapy before surgery	
yp 0	7/29 (24.1%)
yp 1	7/29 (24.1%)
yp 2	10/29 (34.5%)
yp 3–4 ^d^	5/29 (17.2%)
Molecular subtype	
HR+/HER2− ^d^	79/132 (59.8%)
HR+/HER2+	12/132 (9.1%)
HR−/HER2+	4/132 (3%)
Tripe-negative	37/132 (28%)
Ki-67 (%)	39.4 ± 29.7
Low, ≤20% ^e^	50/132 (37.9%)
High, >20% ^e^	82/132 (62.1%)

Data are presented as mean ± standard deviation or number/total number (percentage); HER2: human epidermal growth factor receptor 2; HR: hormone receptor. ^a^ The total number is >132 because two cases were included (one ^b^ with invasive and metaplastic carcinoma and another ^c^ with mucinous and papillary carcinoma) with duplications. ^d^ Included two patients with metastasis observed at the time of diagnosis. ^e^ Included one of two patients with metastasis observed at the time of diagnosis. ^f^ Calculated among the available examinations.

**Table 3:** Mammographic findings of 111 young patients with breast cancer.

Variables	Value
Mammographic breast density	
Fatty breast	3/111 (2.7%)
Dense breast ^a^	108/111 (97.3%)
Lesion type	
Negative	11/111 (9.9%)
Mass	34/111 (30.6%)
Mass and calcifications ^a^	44/111 (39.6%)
Calcifications only	12/111 (10.8%)
Asymmetry	8/111 (7.2%)
Asymmetry with calcifications	2/111 (1.8%)
Mass (n = 78) ^a^	
Shape	
Oval/round ^a^	20/78 (25.6%)
Irregular	58/78 (74.4%)
Margin	
Circumscribed	8/78 (10.3%)
Not circumscribed ^a^	70/78 (89.7%)
Density	
Hyper ^b^	26/78 (33.3%)
Iso ^b^	52/78 (66.7%)
Hypo	
Calcifications (n = 58)	
Distribution	
Segmental ^b^	22/58 (37.9%)
Grouped ^b^	18/58 (31.0%)
Regional	16/58 (27.6%)
Diffuse	2/58 (3.5%)
Shape	
Fine linear/pleomorphic ^b^	35/58 (60.3%)
Coarse heterogenous	5/58 (8.6%)
Amorphous ^b^	18/58 (31.0%)
Combined architectural distortion ^a,c^	25/100 (25.0%)

Data are presented as number/total number (percentage). ^a^ Included two patients with metastasis observed at the time of diagnosis. ^b^ Included one of two patients with metastasis observed at the time of diagnosis. ^c^ Calculated from the remainder after excluding 11 negative results.

**Table 4:** Ultrasonographic findings of 112 young patients with breast cancer.

Variables	Value
Background echotexture	
Homogenous-fatty	-
Homogenous-fibroglandular ^a^	94/112 (83.9%)
Heterogenous	18/112 (16.1%)
Lesion type	
Negative	3/112 (2.7%)
Mass ^a^	90/112 (80.4%)
Non-mass	19/112 (17.0%)
Mass (n = 90) ^a^	
Shape	
Oval/round ^b^	39/90 (44.3%)
Irregular ^b^	51/90 (56.7%)
Orientation	
Parallel ^a^	69/90 (76.7%)
Nonparallel	21/90 (23.3%)
Margin	
Circumscribed	6/90 (6.7%)
Not circumscribed ^a^	84/90 (93.3%)
Echogenicity	
Hypoechoic ^a^	80/90 (88.9%)
Isoechoic	1/90 (1.1%)
Hyperechoic	1/90 (1.1%)
Complexed cystic and solid	5/90 (5.6%)
Heterogeneous	3/90 (3.3%)
Echogenic rind ^b^	19/90 (21.1%)
Non-mass (n = 19)	
Distribution	
Focal	2/19 (10.5%)
Linear/segmental	13/19 (68.4%)
Regional/diffuse	4/19 (21.1%)
Echogenicity	
Hypoechoic	18/19 (94.7%)
Isoechoic	1/19 (5.3%)
Intralesional cysts	3/19 (15.8%)
Calcifications in the lesion ^a,c^	59/109 (54.1%)
Architectural distortion ^b,c^	7/109 (6.4%)
Ductal change ^c^	17/109 (15.6%)
Posterior feature ^c^	
No	79/109 (72.5%)
Enhancement	20/109 (18.3%)
Shadowing	10/109 (9.2%)
Doppler study (n = 50) ^d^	
Avascular	14/50 (28.0%)
Mild ^b^	20/50 (40.0%)
Hypervascular	16/50 (32.0%)

Data are presented as number/total number (percentage). ^a^ Included two patients with metastasis observed at the time of diagnosis. ^b^ Included one of two patients with metastasis observed at the time of diagnosis. ^c^ Calculated from the remainder after excluding three negative results. ^d^ Calculated among the available examinations.

**Table 5:** Magnetic resonance imaging findings of 109 young patients with breast cancer.

Variables	Value
Background parenchymal enhancement	
Minimal to mild ^a^	68/109 (62.4%)
Moderate to marked ^a^	41/109 (37.6%)
Lesion type	
Mass ^b^	91/109 (83.5%)
Non-mass enhancement	18/109 (16.5%)
Mass (n = 91) ^b^	
Shape	
Oval/round ^a^	50/91 (55%)
Irregular ^a^	41/91 (45.1%)
Margin	
Circumscribed	9/91 (9.9%)
Not circumscribed ^b^	82/91 (90.1%)
Enhancement pattern	
Homogenous	26/91 (28.6%)
Heterogenous ^b^	65/91 (71.4%)
Rim enhancement ^a^	59/91 (64.8%)
T2 high signal intensity ^b^	18/91 (19.8%)
Non-mass enhancement (n = 18)	
Distribution	
Focal	-
Segmental	10/18 (55.6%)
Regional	1/18 (5.6%)
Diffuse	7/18 (38.9%)
Enhancement pattern	
Homogenous	7/18 (38.9%)
Heterogenous	11/18 (61.1%)
Enhancement kinetics	
Persistent	7/109 (6.4%)
Plateau	23/109 (21.1%)
Washout ^b^	79/109 (72.5%)

Data are presented as number/total number (percentage). ^a^ Included one of two patients with metastasis observed at the time of diagnosis. ^b^ Included two patients with metastasis observed at the time of diagnosis.

**Table 6:** Statistically significant factors associated with recurrence and survival in the follow-up data of 130 young patients with breast cancer.

Variables	Recurrence				Survival		
	Non (n = 93)	Recurrence (n = 37)	*p*		Survive (n = 115)	Death (n = 15)	*p*
Median body mass index, kg/m^2^	20.33	20.93	0.352 ^a^		20.33	21.57	0.040 ^a^
*BRCA* gene mutation test (n = 54) ^b^			0.617 ^c^				0.046 ^c^
*BRCA1*	2/28 (7.1)	4/28 (15.4)			3/47 (6.4)	3/7 (42.9)	
*BRCA2*	3/28 (10.7)	2/28 (7.7)			5/47 (10.6)	-	
Negative	23/28 (82.1)	20/28 (76.9)			39/47 (83.0)	4/7 (57.1)	
With preoperative NAC (n = 27)	18/93 (19.4)	9/37 (24.3)	0.513 ^c^		20/115 (17.4)	7/15 (46.7)	0.016 ^c^
Total mastectomy (n = 33)			0.805				0.044 ^c^
Without ALND	11/22 (50.0)	5/11 (45.5)			16/28 (57.1)	-	
With ALND	11/22 (50.0)	6/11 (54.6)			12/28 (42.9)	5/5 (100.0)	
Additional hormone therapy after surgery	68/93 (73.1)	21/37 (56.8)	0.109		83/115 (72.2)	6/15 (40.0)	0.018
Recurrence					24/115 (20.9)	13/15 (86.7)	<0.001 ^c^
Local					10/115 (8.7)	9/15 (69.2)	0.209 ^c^
Metachronous contralateral breast					7/115 (6.1)	-	0.038 ^c^
Distant metastasis					8/115 (7.0)	11/15 (73.3)	0.008
Death (n = 15)	2/93 (2.2)	13/37 (35.1)	<0.001 ^c^				
Mean tumor size (cm)	3.40 ± 2.25	3.25 ± 2.04	0.728 ^d^		3.19 ± 2.04	4.63 ± 2.86	0.016 ^d^
Histologic grade of invasive ^b^			0.195				0.018
Well differentiated	20/73 (27.4)	4/34 (11.8)			24/93 (25.8)	-	
Moderately differentiated	29/73 (39.7)	16/34 (47.1)			40/93 (43.0)	5/14 (35.7)	
Poorly differentiated	24/73 (32.9)	14/34 (41.2)			29/93 (31.2)	9/14 (64.3)	
Positive progesterone receptor	62/93 (66.7)	20/37 (54.1)	0.253		79/115 (68.7)	3/15 (20.0)	0.001
Ki-67					36.3 ± 29.2	63.3 ± 21.4	0.001 ^d^
≤20%	40/93 (43.0)	9/37 (24.3)	0.075 ^c^		49/115 (42.6)	-	0.001 ^c^
>20%	53/93 (57.0)	28/37 (75.7)			66/115 (57.4)	15/15 (100.0)	
Ultrasound							
Mass—no echogenic rind (n = 88)	44/61 (40.0)	26/27 (96.3)	0.009 ^c^		60/77 (77.9)	10/11 (90.9)	0.448 ^c^

Data are presented as mean ± standard deviation or number/total number (percentage); NAC: neoadjuvant chemotherapy; ALND: axillary lymph node dissection. ^a^ Wilcoxon rank sum test was applied. ^b^ Calculated among the available examinations. ^c^ Fisher’s exact test was applied. ^d^ Independent *t*-test was applied.

**Table 7:** Statistically significant factors in Cox proportional hazards regression analysis of the disease-free and overall survival of 130 young patients with breast cancer.

Variables	DFS				OS		
	Hazard Ratio	95% CI	*p*		Hazard Ratio	95% CI	*p*
Positive *BRCA1* gene mutation	3.90	1.31–11.62	0.014		10.09	2.20–46.21	0.003
With preoperative neoadjuvant chemotherapy	1.29	0.61–2.72	0.513		3.62	1.31– 10.00	0.013
Additional hormone therapy after surgery	0.54	0.28–1.03	0.062		0.28	0.10–0.78	0.015
Recurrence							
None	-				1 (reference)		
Present					18.58	4.19–82.40	<0.001
Local					19.73	6.26–62.21	<0.001
Metachronous contralateral breast					N/A		-
Distant metastasis					40.42	8.92–183.12	<0.001
Death	33.44	12.81–87.31	<0.001		-		
Tumor size	0.99	0.85–1.16	0.930		1.23	1.04–1.45	0.017
Positive estrogen receptor	0.84	0.43–1.64	0.610		0.33	0.12–0.94	0.038
Positive progesterone receptor	0.63	0.33–1.21	0.164		0.13	0.04–0.48	0.002
Mean Ki-67	1.01	1.00–1.02	0.229		1.03	1.01–1.05	0.003
High Ki-67 (>20%)	2.04	0.96–4.33	0.063		N/A		
Mammographic lesion type							
Mass	1 (reference)				1 (reference)		
Asymmetry with calcifications	4.77	1.02–22.23	0.047		4.58	0.51–41.12	0.174
Ultrasound							
Mass—no echogenic rind	8.37	1.13–61.70	0.037		2.75	0.35–21.48	0.335
Doppler							
Negative	1 (reference)				1 (reference)		
Mild	5.35	1.02–28.09	0.048		N/A		

CI: confidence interval; DFS: disease-free survival; N/A: not available; OS: overall survival.

## Data Availability

The data supporting the findings of this study are available upon reasonable request from the corresponding author.

## Supplementary Materials

The following supporting information can be downloaded at https://www.mdpi.com/article/10.3390/diagnostics15162072/s1, Table S1: Follow-up data without statistical significance in the clinicopathologic characteristics of 130 young patients with breast cancer; Table S2: Follow-up data without statistical significance in the radiologic findings of 110 available young patients with breast cancer, including three pregnant patients; Table S3: Cox proportional hazards regression model analysis of disease-free and overall survival for basic characteristics without statistical significances in clinicopathologic factors; Table S4: Cox proportional hazards regression model analysis of disease-free and overall survival for radiological findings without statistical significance.

## Abbreviations

The following abbreviations are used in this manuscript:

BC	Breast cancer
BMI	Body mass index
CI	Confidence interval
CTX	Chemotherapy
DFS	Disease-free survival
EIC	Extensive intraductal component
ER	Estrogen receptor
HER2	Human epidermal growth factor receptor 2
HR	Hormone receptor
HRT	Hormone therapy
LVI	Lymphovascular invasion
MRI	Magnetic resonance imaging
NAC	Neoadjuvant chemotherapy
OS	Overall survival
pCR	Pathologic complete response
PR	Progesterone receptor
RTx	Radiation therapy
TNBC	Triple-negative breast cancer
US	Ultrasound
