# Supplementary material for: Prognosis of Breast Cancer in Women in Their 20s: Clinical and Radiological Insights
Source: Diagnostics (Basel). 2025 Aug 19;15(16):2072. doi: 10.3390/diagnostics15162072 (PMC12386017; doi:10.3390/diagnostics15162072)
Supplement: Supplementary file 1 [file diagnostics-15-02072-s001.zip › diagnostics-3781530-supplementary.pdf]

Table S1. Follow-up data without statistical significance in the clinicopathologic characteristics of 130 young patients with breast cancer

Variables	Recurrence			Survival		
	Non (n = 93)	Recurrence (n = 37)	*p*-Value	Survive (n = 115)	Death (n = 15)	*p*-Value
Mean age (years)	27.3 ± 1.9	26.8 ± 2.0	0.241	27.7 ± 1.9	26.9 ± 1.9	0.582
With Family history	13 (14.0)	6 (16.2)	0.959	17 (14.8)	2 (13.3)	> 0.999 [a]
Pathologic CR after NAC (n = 27)	4 (22.2)	-	0.267 [a]	4 (20.0)	-	0.545 [a]
Method of surgery			0.621			0.529 [a]
Breast conserving surgery	71 (76.3)	26 (70.3)	0.844	87 (75.7)	10 (66.7)	>0.999 [a]
Without ALND	43 (60.6)	17 (65.4)		54 (62.1)	6 (60.0)	
With ALND	28 (39.4)	9 (34.6)		33 (37.9)	4 (40.0)	
Total mastectomy	22 (23.7)	11 (29.7)		28 (24.4)	5 (33.3)	
Additional therapy after surgery						
Radiation therapy	72 (77.4)	28 (75.7)	>0.999	89 (77.4)	11 (73.3)	0.748 [a]
Chemotherapy	50 (53.8)	25 (67.6)	0.215	68 (59.1)	7 (46.7)	0.521
Histopathologic result			0.659			0.545
Invasive carcinoma, no special type	71 (75.5)	32 (86.5)	> 0.999	90 (76.9)	13 (86.7)	> 0.999
Ductal carcinoma *in situ*	10 (10.6)	2 (5.4)	> 0.999	12 (10.3)	-	> 0.999
Mucinous carcinoma	7 (7.5)	-	> 0.999	7 (6.0)	-	> 0.999
Metaplastic carcinoma	2 (2.1)	2 (5.4)	> 0.999	3 (2.6)	1 (6.7)	> 0.999
Papillary carcinoma	1 (1.1)	1 (2.7)	> 0.999	2 (1.7)	-	> 0.999
Invasive lobular carcinoma	1 (1.1)	1 (2.7)	> 0.999	1 (0.9)	1 (6.7)	0.914
Medullary carcinoma	1 (1.1)	-	> 0.999	1 (0.9)	-	> 0.999
Secretary carcinoma	1 (1.1)	-	> 0.999	1 (0.9)	-	> 0.999
With Multiplicity	17 (18.3)	11 (29.7)	0.231	25 (21.7)	3 (20.0)	> 0.999 [a]
With Lymphovascular invasion [b]	39 (47.0)	13 (37.1)	0.369	45 (43.3)	7 (46.7)	> 0.999
With Extensive intraductal component [b]	26 (35.1)	13 (37.1)	> 0.999	35 (36.8)	4 (28.6)	0.761
With Nipple areolar complex involvement	6 (6.5)	3 (8.1)	0.713 [a]	8 (7.0)	1 (6.7)	> 0.999 [a]
Surgical staging without NAC			0.699 [a]			0.408 [a]
Stage 0	10 (13.3)	2 (7.1)		12 (12.6)	-	
Stage 1	29 (38.7)	11 (39.3)		38 (40.0)	2 (25.0)	
Stage 2	32 (42.7)	12 (42.9)		39 (41.1)	5 (62.5)	

Stage 3-4	4 (5.3)	3 (10.7)		6 (6.3)	1 (12.5)	
Surgical staging with NAC (n = 27) [b]			0.097			0.067
yp0/CR	7 (38.9)	-		7 (35.0)	-	
yp1	5 (27.8)	2 (22.2)		6 (30.0)	1 (14.3)	
yp2	5 (27.8)	5 (55.6)		6 (30.0)	4 (57.1)	
yp3-4	1 (5.6)	2 (22.2)		1 (5.0)	2 (28.6)	
Positive ER	62 (66.7)	23 (62.2)	0.777	79 (68.7)	6 (40.0)	0.056
Positive HER2	12 (12.9)	5 (13.5)	> 0.999	14 (12.2)	3 (20.0)	0.415 [a]
Positive HR (ER or PR positive)	66 (71.0)	23 (62.2)	0.440	82 (71.3)	7 (46.7)	0.075 [a]
Molecular subtype			0.820 [a]			0.193 [a]
HR+/HER2-	57 (61.3)	20 (54.1)		71 (61.7)	6 (40.0)	
HR+/HER2+	9 (9.7)	3 (8.1)		11 (9.6)	1 (6.7)	
HR-/HER2+	3 (3.2)	1 (2.7)		3 (2.6)	1 (6.7)	
Tripe negative	24 (25.8)	13 (35.1)		30 (26.1)	7 (46.7)	

(Data presented as Mean ± SD or n (%)); pCR : pathologic complete response; NAC : neoadjuvant chemotherapy; ALND : axillary lymph node dissection; ER : estrogen receptor; HER2 : human epidermal growth factor receptor 2; HR : hormone receptor; PR : progesterone receptor.

[a] Fisher's exact test was applied.

[b] Calculated among the available examinations.

Table S2. Follow-up data without statistical significance in the radiologic findings of 110 available young patients with breast cancer, including three pregnant patients

2-1. Mammographic findings of 109 available patients.

Variables	Recurrence			Survival		
	Non (n = 78)	Recurrence (n = 31)	*p*-Value	Survive (n = 95)	Death (n = 14)	*p*-Value
Mammographic breast density			> 0.999 [a]			0.341 [a]
Fatty breast	2 (2.6)	1 (3.2)		2 (2.1)	1 (7.1)	
Dense breast	76 (97.4)	30 (96.8)		93 (97.9)	13 (98.9)	
Lesion type			0.247 [a]			0.538 [a]
Negative	10 (12.8)	1 (3.2)		10 (10.5)	1 (7.1)	
Mass	25 (32.1)	9 (29.0)		30 (31.6)	4 (28.6)	
Mass with calcifications	29 (37.2)	13 (41.9)		35 (36.8)	7 (50.0)	
Calcification only	8 (10.3)	4 (12.9)		11 (11.6)	1 (7.1)	
Asymmetry	6 (7.7)	2 (6.5)		8 (8.4)	-	
Asymmetry with calcifications	-	2 (6.5)		1 (1.1)	1 (7.1)	
Mass (*N* = 76)						
Shape			0.868			> 0.999 [a]
Oval/round	15 (27.8)	5 (22.7)		17 (26.2)	3 (27.3)	
Irregular	39 (72.2)	17 (77.3)		48 (73.8)	8 (72.7)	
Margin			0.42 5[a]			0.594 [a]
Circumscribed	7 (13.0)	1 (4.5)		8 (12.3)	-	
Not circumscribed	47 (87.0)	21 (95.5)		57 (87.7)	11 (100.0)	
Density			0.806 [a]			0.193 [a]
Hyper	19 (31.7)	9 (34.6)		22 (29.7)	6 (50.0)	
Iso	41 (68.3)	17 (65.4)		52 (70.3)	6 (50.0)	
Hypo	-	-		-	-	
Calcifications (n = 56)						
Distribution			0.250 [a]			0.809 [a]
Segmental	15 (40.5)	6 (31.6)		18 (38.3)	3 (33.3)	
Grouped	8 (21.6)	9 (47.4)		13 (27.7)	4 (44.4)	
Regional	12 (32.4)	4 (21.1)		14 (29.8)	2 (22.2)	

Diffuse	2 (5.4)	-		2 (4.3)	-	
Shape			0.564 [a]			> 0.99 9[a]
Fine linear/pleomorphic	21 (56.8)	13 (68.4)		29 (61.7)	5 (55.6)	
Coarse heterogenous	3 (8.1)	2 (10.5)		4 (8.5)	1 (11.1)	
Amorphous	13 (35.1)	4 (21.1)		14 (29.8)	3 (33.3)	
Architectural distortion [b]	17 (25.0)	6 (20.0)	0.751	20 (23.5)	3 (23.1)	> 0.999 [a]

[a] Fisher's exact test was applied.

[b] Calculated from the remainder after excluding 11 negative results.

2-2. Ultrasound findings of 110 patients.

Variables	Recurrence			Survival		
	Non (n = 79)	Recurrence (n = 31)	*p*-Value	Survive (n = 96)[a]	Death (n = 14)	*p*-Value
Background echotexture			0.579 [a]			0.240 [a]
Homogenous-fatty	-	-		-	-	
Homogenous-fibroglandular	67 (84.8)	25 (80.6)		82 (85.4)	10 (71.4)	
Heterogenous	12 (15.2)	6 (19.4)		14 (14.6)	4 (28.6)	
Lesion type			0.520 [a]			> 0.809 [a]
Negative	3 (3.8)	-		3 (3.1)	-	
Mass	61 (77.2)	27 (87.1)		77 (80.2)	11 (78.6)	
Nonmass	15 (19.0)	4 (12.9)		16 (16.7)	3 (21.4)	
Mass (n = 88)						
Shape			0.941			> 0.999 [a]
Oval/round	27 (44.3)	11 (40.7)		33 (42.9)	5 (45.5)	
Irregular	34 (55.7)	16 (59.3)		44 (57.1)	6 (54.5)	
Orientation			0.292			> 0.999 [a]
Parallel	44 (72.1)	23 (85.2)		58 (75.3)	9 (81.8)	
Nonparallel	17 (27.9)	4 (14.8)		19 (24.7)	2 (18.2)	
Margin			> 0.999 [a]			> 0.563 [a]
Circumscribed	4 (6.6)	2 (7.4)		5 (6.5)	1 (9.1)	
Not circumscribed	57 (93.4)	25 (92.6)		72 (93.5)	10 (90.9)	
Echogenicity			0.801 [a]			0.357 [a]
Hypoechoic	53 (86.9)	25 (92.6)		69 (89.6)	9 (81.8)	
Isoechoic	1 (1.6)	-		1 (1.3)	-	
Hyperechoic	1 (1.6)	-		1 (1.3)	-	
Complexed cystic and solid	3 (4.9)	2 (7.4)		3 (3.9)	2 (18.2)	
Heterogeneous	3 (4.9)	-		3 (3.9)	-	
Non-mass (n = 19)						
Distribution			> 0.999 [a]			0.678 [a]
Focal	2 (13.3)	-		2 (12.5)	-	
Linear/segmental	10 (66.7)	3 (75.0)		10 (62.5)	3 (100.0)	

Regional/Diffuse	3 (20.0)	1 (25.0)		4 (25)	-	
Echogenicity			> 0.999 [a]			> 0.999 [a]
Hypoechoic	14 (93.3)	4 (100.0)		15 (93.8)	3 (100.0)	
Isoechoic	1 (6.7)	-		1 (6.3)	-	
Intralesional cysts	2 (2.5)	1 (3.2)	0.590 [a]	3 (3.1)	-	> 0.999 [a]
Calcifications in the lesion [b]	38 (50)	19 (61.3)	0.396	48 (51.6)	9 (64.3)	0.549
Architectural distortion [b]	5 (6.6)	1 (3.2)	0.670 [a]	5 (5.4)	1 (7.1)	0.578 [a]
Ductal change [b]	10 (13.2)	7 (22.6)	0.251 [a]	15 (16.1)	2 (14.3)	> 0.999 [a]
Posterior feature [b]			0.302 [a]			0.272 [a]
No	57 (75.0)	20 (64.5)		68 (73.1)	9 (64.3)	
Enhancement	14 (18.4)	6 (19.4)		18 (19.4)	2 (21.4)	
Shadowing	5 (6.6)	5 (16.1)		7 (7.5)	3 (50.0)	
Doppler [c]			0.193			> 0.999 [a]
Avascular	12 (36.4)	2 (12.5)		14 (33.3)	-	
Mild	10 (30.3)	8 (53.3)		15 (35.7)	3 (50.0)	
Hypervascular	11 (33.3)	5 (31.3)		13 (31.0)	3 (50.0)	

[a] Fisher's exact test was applied.

[b] Calculated from the remainder after excluding 3 negative results.

[c] Calculated among the available examinations.

2-3. MRI findings of 107 patients.

Variables	Recurrence			Survival		
	**Non (n = 76)**	**Recurrence (n = 31)**	***p*-Value**	**Survive (n = 93)**	**Death (n = 14)**	***p*-Value**
Background parenchymal enhancement			0.120			0.179
Minimal to mild	51 (67.1)	16 (51.6)		61 (65.6)	6 (42.9)	
Moderate to marked	25 (32.9)	15 (48.4)		32 (34.4)	8 (57.1)	
Lesion Type			0.580 [a]			0.248 [a]
Mass	62 (81.6)	27 (87.1)		79 (84.9)	10 (71.4)	
Nomass enhancement	14 (18.4)	4 (12.9)		14 (15.1)	4 (28.6)	
Mass (n = 89)						
Shape			0.866			0.749 [a]
Oval/round	35 (56.5)	14 (51.9)		44 (55.7)	5 (50.0)	
Irregular	27 (43.5)	13 (48.1)		35 (44.3)	5 (50.0)	
Margin			0.717 [a]			> 0.999 [a]
Circumscribed	7 (11.3)	2 (7.4)		8 (10.1)	1 (10.0)	
Not circumscribed	55 (88.7)	25 (92.6)		71 (89.9)	9 (90.0)	
Enhancement pattern			0.226			0.717 [a]
Homogenous	21 (33.9)	5 (18.5)		24 (30.4)	2 (20.0)	
Heterogenous	41 (66.1)	22 (81.5)		55 (69.6)	8 (80.0)	
Rim enhancement	40 (64.5)	18 (66.7)	> 0.999	50 (63.3)	8 (80.0)	0.484 [a]
T2 high signal intensity	14 (22.6)	2 (7.4)	0.133	16 (20.3)	-	0.198
Nonmass enhancement (n = 18)						
Distribution			> 0.999 [a]			> 0.999 [a]
Focal	-	-		-	-	
Segmental	8 (57.1)	2 (50.0)		8 (57.1)	2 (50.0)	
Regional	1 (7.1)	-		1 (7.1)	-	
Diffuse	5 (35.7)	2 (50.0)		5 (35.7)	2 (50.0)	
Enhancement pattern			> 0.999 [a]			0.119 [a]
Homogenous	6 (42.9)	1 (25.0)		7 (50.0)	-	
Heterogenous	8 (57.1)	3 (75.0)		7 (50.0)	4 (100.0)	
Enhancing kinetics			0.497 [a]			0.888 [a]

Persistent	6 (7.9)	1 (3.2)	6 (6.5)	1 (7.1)
Plateau	18 (23.7)	5 (16.1)	21 (22.6)	2 (14.3)
Wash-out	52 (68.4)	25 (80.6)	66 (71.0)	11 (78.6)

[a] Fisher's exact test was applied.

Table S3. Cox proportional hazards regression model analysis of disease-free and overall survival for basic characteristics without statistical significances in clinicopathologic factors

Variables	Disease-free survival			Overall survival		
	Hazard ratio	95% CI	*p*-Value	Hazard ratio	95% CI	*p*-Value
With Family history	1.23	0.51–2.96	0.642	0.98	0.22–4.32	0.973
Median body mass index, kg/m^2	1.14	0.98–1.31	0.082	1.04	0.94–1.10	0.475
Method of surgery			0.535			0.741
Breast conserving surgery	1 (reference)		0.433	1 (reference)		0.486
Without ALND	1 (reference)			1 (reference)		
With ALND	0.72	0.32–1.63		1.01	0.29–3.59	0.985
Total mastectomy	1.25	0.62–2.53	0.677	1.41	0.48–4.13	0.528
Without ALND	1 (reference)			1 (reference)		
With ALND	1.29	0.39–4.24		N/A		
Additional therapy after surgery						
Radiation therapy	0.82	0.39–1.73	0.601	0.79	0.25–2.49	0.693
Chemotherapy	1.37	0.68–2.74	0.377	0.6	0.22–1.66	0.325
Histopathologic result						
Invasive carcinoma, no special type	1 (reference)			1 (reference)		
Ductal carcinoma *in situ*	0.63	0.15–2.64	0.529	N/A		
others	0.81	0.29–2.31	0.699	0.1	0.22–4.42	0.997
Multiplicity	1.42	0.70–2.88	0.328	0.83	0.23–2.94	0.773
Lymphovascular invasion	0.62	0.31–1.23	0.173	1.09	0.40–3.01	0.865
Extensive intraductal component	1.11	0.56–2.21	0.763	0.68	0.21–2.18	0.522
Nipple areolar complex involvement	1.12	0.34–3.66	0.847	0.94	0.12–7.12	0.949
Histologic grade of invasive cancer						
well	0.43	0.14–1.32	0.140	N/A		
moderate	0.95	0.46–1.95	0.891	0.44	0.15–1.31	0.140
poorly	1 (reference)			1 (reference)		
Surgical staging without NAC						
Stage 0	0.44	0.07–2.62	0.364	N/A		
Stage 1	0.6	0.17–2.15	0.431	0.34	0.03–3.69	0.372

Stage 2	0.58	0.16–2.07	0.403	0.77	0.09–6.57	0.809
Stage 3-4	1 (reference)			1 (reference)		
Surgical staging with NAC (n = 27)						
yp0/CR	N/A			N/A		
yp1	0.25	0.03–1.80	0.169	0.18	0.02–1.96	0.158
yp2	0.53	0.1–2.76	0.452	0.55	0.1–3.05	0.495
yp3-4	1 (reference)			1 (reference)		
Positive HER2	1.14	0.44–2.93	0.784	1.63	0.46–5.78	0.449
Positive HR (ER or PR positive)	0.76	0.39–1.48	0.418	0.4	0.14–1.1	0.075
High Ki-67 (>20%)	2.04	0.96–4.33	0.063	N/A		
Molecular subtype						
HR+/HER2-	1 (reference)			1 (reference)		
HR+/HER2+	0.98	0.29–3.29	0.969	0.95	0.11–7.88	0.961
HR-/HER2+	1.03	0.14–7.71	0.975	3.34	0.40–27.76	0.264
Tripe negative	1.34	0.67–2.70	0.412	2.41	0.81–7.17	0.114

CI : confidence interval; ALND : axillary lymph node dissection; N/A : not available; NAC : neoadjuvant chemotherapy; HER2 : human epidermal growth factor receptor 2; HR : hormone receptor; ER : estrogen receptor; PR : progesterone receptor.

Table S4. Cox proportional hazards regression model analysis of disease-free and overall survival for radiological findings without statistical significance

Variables	Disease-free survival			Overall survival		
	Hazard ratio	95% CI	*p*-Value	Hazard ratio	95% CI	*p*-Value
Mammography						
Mammographic breast density						
Fatty breast	1 (reference)			1 (reference)		
Dense breast	0.69	0.09–5.12	0.720	0.36	0.05–2.75	0.325
Lesion type						
Mass	1 (reference)			1 (reference)		
Mass with calcifications	1.19	0.51–2.79	0.948	1.39	0.41–4.76	0.597
Calcification only	1.09	0.34–3.56	0.882	0.62	0.07–5.54	0.668
Asymmetry	0.78	0.17–3.59	0.744	N/A		
Mass						
Shape						
Oval/round	1 (reference)			1 (reference)		
Irregular	1.00	0.36–2.75	0.998	0.90	0.24–3.40	0.878
Margin						
Circumscribed	1 (reference)			1 (reference)		
Not circumscribed	2.17	0.29–16.21	0.451	N/A		
Density						
Hyperdense	1 (reference)			1 (reference)		
Isodense	0.85	0.38–1.91	0.693	0.46	0.15–1.43	0.181
Hypodense	N/A			N/A		
Calcifications						
Distribution						
Segmental	1 (reference)			1 (reference)		
Grouped	2.17	0.77–6.12	0.142	1.84	0.41–8.21	0.427
Regional	0.82	0.23–2.93	0.765	0.97	0.16–5.81	0.973
Diffuse	N/A			N/A		
Shape						

Fine linear/pleomorphic	1 (reference)			1 (reference)		
Coarse heterogenous	1.44	0.32–6.44	0.634	1.57	0.18–13.51	0.679
Amorphous	0.56	0.18–1.74	0.317	1.33	0.32–5.57	0.697
Architectural distortion	0.83	0.34–2.03	0.684	0.98	0.27–3.56	0.974
Ultrasound						
Background echotexture						
Homogenous fatty	N/A			N/A		
Homogenous fibroglandular	1 (reference)			1 (reference)		
Heterogenous	1.46	0.60–3.58	0.405	2.20	0.69–7.01	0.183
Lesion type						
Mass	1 (reference)			1 (reference)		
Nonmass	0.69	0.24–1.98	0.494	1.23	0.34–4.43	0.746
Negative	N/A			N/A		
Mass						
Shape						
Oval/round	1 (reference)			1 (reference)		
Irregular	1.02	0.47–2.21	0.953	0.82	0.25–2.69	0.744
Orientation						
Parallel	1 (reference)			1 (reference)		
Nonparallel	0.48	0.16–1.42	0.185	0.67	0.15–3.12	0.614
Margin						
Circumscribed	1 (reference)			1 (reference)		
Not circumscribed	0.92	0.22–3.90	0.913	0.76	0.10–5.95	0.795
Echogenicity						
Hypoechoic	1 (reference)			1 (reference)		
Isoechoic	N/A			N/A		
Hyperechoic	N/A			N/A		
Complexed cystic and solid	1.75	0.41–7.46	0.447	3.99	0.86–18.48	0.077
Heterogeneous	N/A			N/A		
Nonmass						
Distribution						
Linear/segmental	1 (reference)			1 (reference)		

Focal/Regional/Diffuse	0.65	0.07-6.22	0.705	N/A		
Intralesional cysts	1.28	0.13–12.39	0.832	N/A		
Calcifications in the lesion	1.37	0.66–2.82	0.395	1.54	0.52–4.60	0.438
Architectural distortion	0.49	0.07–3.63	0.488	1.36	0.18–10.39	0.768
Ductal change	1.73	0.74–4.04	0.208	0.79	0.18–3.51	0.753
Posterior feature						
No	1 (reference)			1 (reference)		
Enhancement	1.17	0.47–2.91	0.740	0.82	0.18–3.81	0.803
Shadowing	2.05	0.76–5.51	0.156	2.88	0.78–10.66	0.112
Doppler						
Avascular	1 (reference)			1 (reference)		
Mild	5.35	1.02–28.09	0.048	N/A		
Hypervascular	2.9	0.54–15.5	0.213	N/A		
MRI						
Background parenchymal enhancement						
Minimal to mild	1 (reference)			1 (reference)		
Moderate to marked	1.97	0.97–4.01	0.061	2.46	0.86–7.18	0.091
Lesion Type						
Mass	1 (reference)			1 (reference)		
Nonmass enhancement	0.82	0.29–2.36	0.719	2.22	0.69–7.07	0.179
Mass						
Shape						
Oval/round	1 (reference)			1 (reference)		
Irregular	1.13	0.53–2.40	0.754	1.23	0.36–4.24	0.745
Margin						
Circumscribed	1 (reference)			1 (reference)		
Not circumscribed	1.23	0.29–5.23	0.777	0.87	0.11–6.85	0.893
Enhancement pattern						
Homogenous	1 (reference)			1 (reference)		
Heterogenous	2.03	0.77–5.37	0.153	1.72	0.36–8.08	0.495
Rim enhancement	1.11	0.50–2.49	0.792	2.33	0.49–10.99	0.284
T2 high signal intensity	0.35	0.08–1.47	0.151	N/A		

Nonmass enhancement						
Distribution						
Focal	N/A			N/A		
Segmental	1 (reference)			1 (reference)		
Regional	N/A			N/A		
Diffuse	1.41	0.2–10.04	0.729	1.40	0.20–9.95	0.739
Enhancement pattern						
Homogenous	1 (reference)			1 (reference)		
Heterogenous	2.56	0.26–24.87	0.418	N/A		
Enhancing kinetics						
Persistent	1 (reference)			1 (reference)		
Plateau	1.57	0.18–13.50	0.684	0.68	0.06–7.46	0.749
Wash-out	2.48	0.33–18.32	0.375	1.07	0.14–8.32	0.945

CI : confidence interval; N/A : not available; MRI : magnetic resonance imaging.
